# Evidence for classic complement activity in neuromyelitis optica 

**DOI:** 10.5414/NP300697

**Published:** 2014-02-26

**Authors:** Melina V. Jones, Karen Fox-Talbot, Michael Levy

**Affiliations:** Johns Hopkins University, Baltimore, MD, USA

**Keywords:** complement activity, neuromyelitis optica

## Abstract

Abstract not available.

Sir, – Neuromyelitis optica is a relapsing-remitting autoimmune disease associated with the anti-aquaporin-4 antibody (NMO-IgG) and complement-mediated perivascular inflammation disease on pathology [1]. Although not specific for NMO, complement deposition is characteristic of the humoral immunopathogenesis. Empiric and laboratory evidence for the involvement of the NMO-IgG as a harmful pathogenic antibody is based on the assumption that the NMO-IgG triggers complement activation via the classical complement pathway [2]. Using a human tissue lesion from an NMO patient, we provide further evidence for the pathogenic antibody model by demonstrating the presence of C4d, a complement component regarded as a sign of an antibody-triggered complement response. 

The autopsy spinal cord tissue was obtained from an NMO-IgG seropositive NMO patient who died shortly after onset of a relapse. Sections were fixed with acetic acid/methanol and stained with anti-C4d (ALPCO Diagnostics, Salem, NH, USA). Figure 1 shows the intense perivascular staining with C4d in this spinal cord lesion (panels A1, A2, and B) compared to a section without the primary C4d antibody (panels C1 and C2). Also provided is an image of humorally mediated heart transplant rejection staining positive for endothelial C4d indicating antibody-mediated classic complement activity (panel D). 

The significance of this image is that two complement inhibitors are being tested for treatment of NMO. Eculizumab (Soliris®), a C5a inhibitor, has been demonstrated to reduce relapses in NMO for at least 1 year [3]. As a downstream target, C5a inhibition covers all complement pathways but it allows some complement components such as C3a to remain active and contribute to inflammation. Purified human C1-esterase inhibitor (Cinryze®) is current being tested for efficacy in acute NMO exacerbations [4]. As an upstream target of the classical pathway, Cinryze may not effectively inhibit the other complement pathways. 

This image suggests that the classical complement pathway is involved in NMO and may thus be responsive to both eculizumab and purified human C1-esterase inhibitor. 

## Conflict of interest 

The authors have no conflicts of interest.

**Figure 1.**
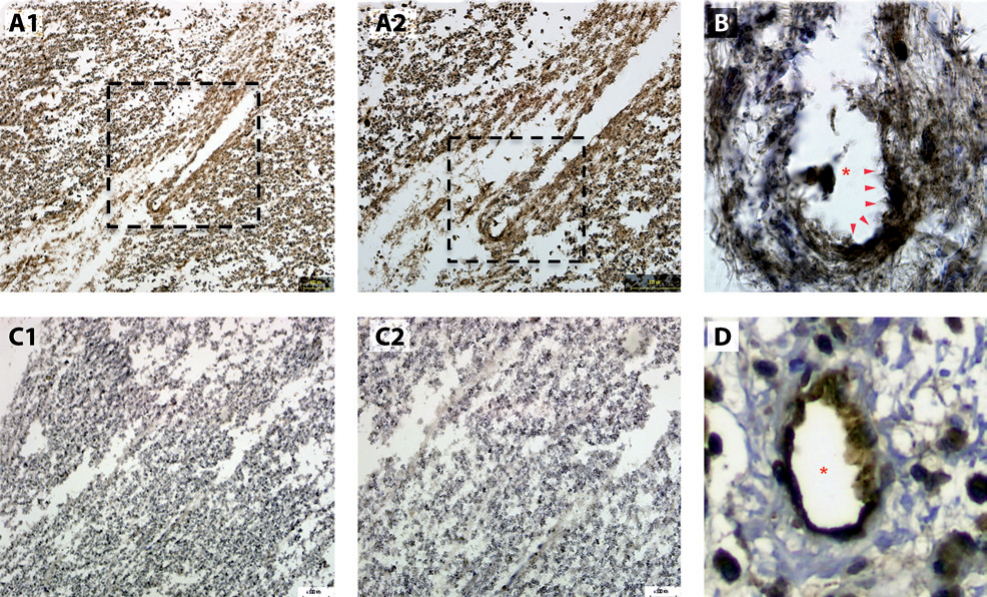
A1: Low power (10 ×) section of autopsied human spinal cord tissue from active lesion in a patient with neuromyelitis optica demonstrating diffuse perivascular c4d complement deposition. A2: Boxed area is magnified in A2. Higher power section (20 ×) of boxed area from A1 showing perivascular C4d staining. B: High power section (40 ×) of boxed area from A2 showing intense perivascular C4d staining (arrows). Asterisk identifies the blood vessel lumen. C1: Low power (10 ×) section of spinal cord without primary antibody against C4d showing counterstain only. C2: Higher power section (20 ×) from C1 confirming no C4d staining. D: Image from transplant rejected heart tissue showing classical perivascular c4d staining pattern. Asterisk identifies the blood vessel lumen.
